# Supplementary figures and images for: Scorpion envenomation in the state of São Paulo, Brazil: Spatiotemporal analysis of a growing public health concern
Source: PLoS One. 2022 Apr 8;17(4):e0266138. doi: 10.1371/journal.pone.0266138 (PMC8992990; doi:10.1371/journal.pone.0266138)

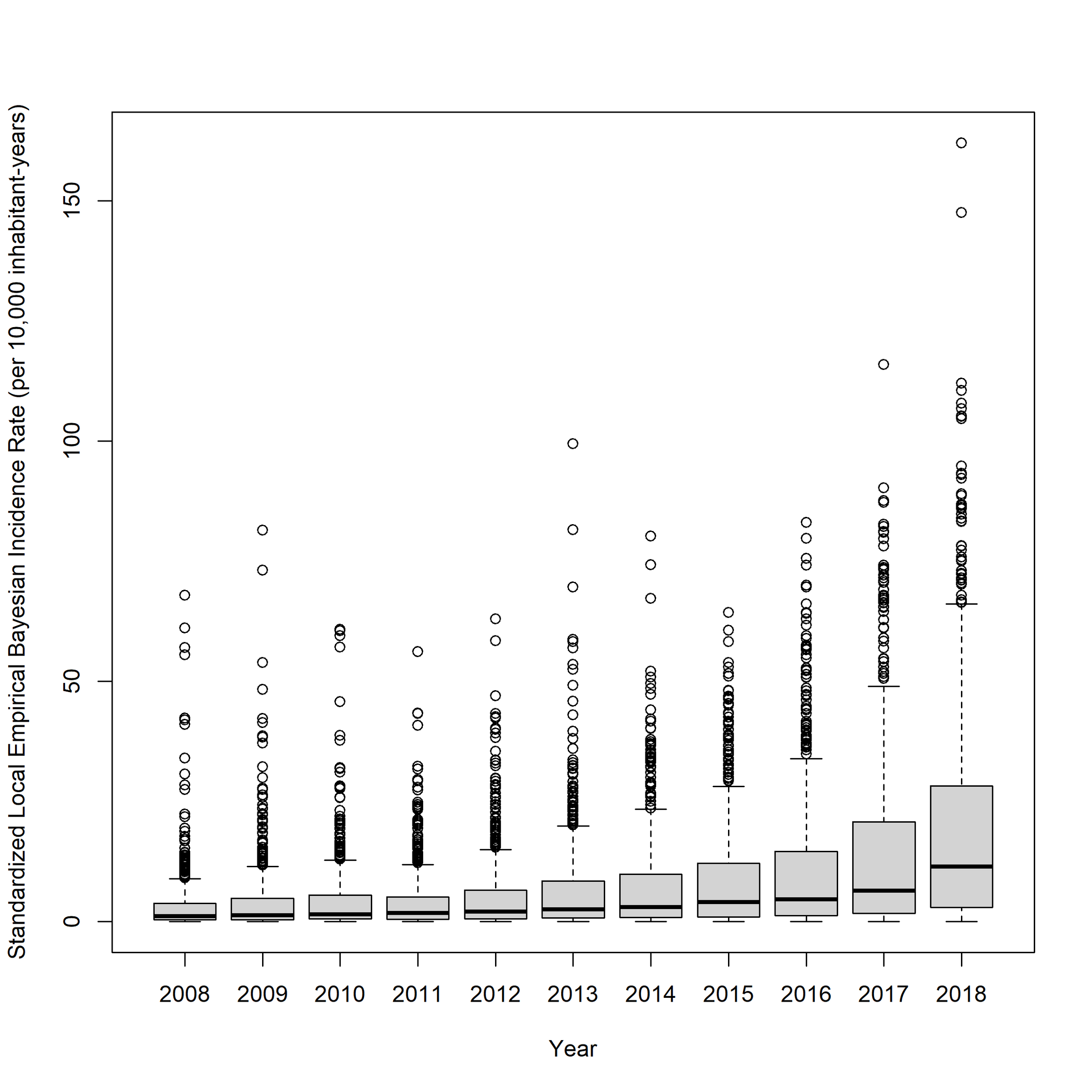

Supplement: S1 Fig — (TIF) [file pone.0266138.s002.tif]
